# Injury Pattern According to Player Position in Male Amateur Football Players in Greece: A Retrospective Study

**DOI:** 10.3390/jcm14176320

**Published:** 2025-09-07

**Authors:** Konstantinos Vassis, Ioannis Misiris, Spyridon Plakias, Athanasios Siouras, Savvas Spanos, Eleftherios Giamouridis, Zacharias Dimitriadis, Dimitrios Tsaopoulos, Ioannis A. Poulis

**Affiliations:** 1 Human Performance and Rehabilitation Laboratory, Faculty of Physiotherapy, School of Health Sciences, University of Thessaly, 35132 Lamia, Greece; sspanos@uth.gr (S.S.); leuteris16@gmail.com (E.G.); ipoulis@uth.gr (I.A.P.); 2 “Physio’clock” Advanced Physiotherapy Center, 41223 Larissa, Greece; ioannis.misiris@gmail.com; 3 Department of Physical Education and Sport Science, University of Thessaly, 42100 Trikala, Greece; spyros_plakias@yahoo.gr; 4 Department of Computer Science and Biomedical Informatics, School of Science, University of Thessaly, 35132 Lamia, Greece; athsiour@gmail.com; 5 Health Assessment and Quality of Life Research Laboratory (HAQL Lab), School of Health Sciences, University of Thessaly, 41110 Lamia, Greece; zdimitriadis@uth.gr; 6 Center for Research and Technology Hellas, Institute for Bio-Economy & Agri-Technology, 60361 Volos, Greece; d.tsaopoulos@certh.gr

**Keywords:** amateur soccer, injury patterns, player position, injury incidence, musculoskeletal injuries

## Abstract

**Background:** Football has a high injury risk due to speed and contact, and injury patterns may vary by playing position. Positional roles affect physical and physiological demands and may influence injury characteristics. Although this has been examined in professionals, data from amateur players are scarce. This study examined the incidence, type, and severity of injuries among amateur footballers in Greece with respect to playing position. **Methods:** A retrospective epidemiological study analyzed musculoskeletal injuries in 222 amateur male football players during the 2022–2023 season. Data were collected via a CHERRIES-compliant online survey (SurveyMonkey^®^) from May to July 2023. Eligible participants were active male athletes aged ≥18 years competing in amateur Greek leagues. Injuries were defined according to the FIFA–UEFA consensus and expressed as incidence rates per 1000 h of exposure. Statistical analyses used SPSS v25 with significance at *p* < 0.05. **Results:** Among players (mean age: 25.3 ± 5.7 years), injury prevalence ranged from 65.1% (DFs) to 79.3% (GKs) with no significant association between playing position and injury risk (*p* = 0.379). Injury incidence ranged from 4.5 to 5.7 per 1000 h, highest among MFs. Incidence rates ranged between 1.33 and 2.74 injuries/1000 h in matches versus 1.33 to 2.09/1000 h in training, with DFs, FWs, and MFs more prone to match injuries, whereas GKs had slightly higher training rates; however, the number of injuries did not significantly differ between games and training across positions (χ^2^ = 5.21, *p* = 0.517). Muscle strains and lower-limb injuries predominated. Injury severity differed significantly by position (*p* = 0.001), but injury type and mechanism did not. **Conclusions:** GKs and MFs showed the highest prevalence and incidence, but position was not linked to overall risk. Severity differences highlight the need for position-specific prevention strategies.

## 1. Introduction

Football is the most widely practiced sport globally, yet it is inherently complex and carries a significant risk of injury as extensively documented in the literature [1]. This injury risk poses a notable socioeconomic burden on elite, youth, and recreational players alike [2]. Recent studies highlight that modern football’s increasing pace demands superior physical performance from players, who face substantial metabolic and biomechanical challenges during matches [3]. The contemporary football landscape is characterized by a growing number of matches each season, often exceeding 70 [4], which adds both physical and mental strain on players [5]. Τhe UEFA Elite Club Injury Study revealed a 2.5-fold increase in training and match time between the 2001/2002 and 2013/2014 seasons, indicating a marked rise in workload [6]. As football evolves, players are likely to experience even higher speeds and denser periods of high-intensity efforts in the future [5].

Given these conditions, football is the sport with the highest number of injuries and the highest injury rate per unit of exposure. Injuries occur during football games and practices due to the combination of high speeds and full contact [7]. According to a recent systematic review [8], injury incidence rates in professional football players (adult athletes) reach 7.75 (5.45–10.03), 3.97 (2.63–5.33), and 30.64 (19.36–40.92) injuries per 1000 h of total, training and match exposure, respectively. For amateur football players, the respective rates are 7.89 (4.94–10.84), 3.22 (1.82–4.62), and 17.56 (11.41–23.71) injuries per 1000 h of exposure. It is also well established that injury risk is significantly higher during matches than training for both professional and amateur players, with match incidence reported as 7.8-times higher among professionals and 5.4-times higher among amateurs [8].

A soccer team comprises 11 players who have different playing positions depending on their location on the pitch and different tactical roles during matches [9]. Positional role appears to influence total energy expenditure in a match, suggesting that players in different positions experience varying physical, physiological, and bioenergetic demands [10,11,12]. Previous research indicates that central defenders and strikers generally cover the least distance during games, whereas central midfielders tend to cover the most distance [13,14]. Conversely, wide players, including both attackers and defenders, record the greatest distances in terms of sprinting and high-intensity running, whereas central players cover the least [15]. The number of accelerations by playing positions follows a similar pattern, with wide-field players accelerating significantly more than central players [16,17]. Additionally, the functional demands related to different football player positions are also significant [18,19,20]. These role-related demands are further mirrored in the anthropometric differences observed among different soccer player positions; notably, forwards typically have a lower body weight and adiposity compared to goalkeepers and defenders [21,22].

As different roles are associated with varying intensity during match play [23] and experience a different combination of anticipated or non-anticipated movement patterns [24], playing position may act as an independent risk factor for injury. The recent literature explores a potential relationship between playing position and injury incidence. Leventer et al. (2016) [25] in their six-season media-based analysis among elite football players in the first division Bundesliga, report that wide midfielders have the highest match injury rate and central DFs the highest training injury incidence rate. Other studies have documented a higher prevalence of injuries in MFs [26] and FWs [27], with lower limbs being the main anatomical locations for these injuries. However, there are divergences in their types, with some studies highlighting muscle damages [28,29,30], while others point to joint and tendon injuries [31] as more frequent. Interestingly, GKs, who engage in less running but more ball reaching and collisions, show a higher rate of upper-extremity, trunk, and head injuries [32].

While the epidemiology of football injuries has been extensively examined in professional settings, research involving amateur players is remarkably limited with regard to injuries and their association with playing position. Only a few studies have focused on this population. Hunt and Fulford (1990) [33], in a hospital-based sample of English amateur footballers, found that most injuries involved the lower limbs and were more frequent among central DFs and FWs, but their data were limited to players who attended an emergency department and did not account for exposure or injury mechanisms. More recently, Onaka et al. (2017) [34] in Brazil, in a mixed sample of professionals and amateurs, reported a higher frequency of injuries among forwards, mostly knee and ankle injuries caused by contact, but did not focus exclusively on amateur athletes and used only simple counts rather than standardized definitions or incidence rates. Similarly, Bello et al. (2020) [35] in Nigeria studied 118 amateur players and reported that most injuries involved the knee and ankle, yet no statistically significant association with playing position was found, and exposure-based rates, injury severity, and preventive aspects were not considered. Overall, these studies present important methodological limitations: they do not use the current FIFA–UEFA consensus definitions, do not quantify exposure or injury incidence, classify positions only broadly, and do not analyze in depth the injury type, severity, mechanisms, or prevention strategies. The present study aims to address these gaps by applying standardized definitions and exposure-based calculations to systematically examine incidence, prevalence, location, type, severity, and mechanisms of injuries according to playing position and by discussing prevention strategies in a large, exclusively amateur, male population in Greece over an entire competitive season.

Despite the limited evidence available, it is clear that injury patterns in amateur football cannot be directly extrapolated from professional settings. Amateur players differ in training volume, fitness, and access to medical care, which may influence both the risk and the mechanisms of injury [36]. Building on this gap, the present study retrospectively investigates injuries in a large cohort of male amateur football players in Greece during a full competitive season. Using standardized FIFA–UEFA definitions and exposure-based calculations, we analyze sthe incidence and prevalence of injuries, detailing their causes, types, locations, and severity, and examining how these factors vary across playing positions. These findings aim to provide a comprehensive understanding of position-specific injury patterns at the amateur level and contribute to more targeted training and prevention strategies.

## 2. Materials and Methods

### 2.1. Study Design

This study is part of a previous retrospective, survey-based epidemiological study investigating musculoskeletal injuries among amateur male football players in Greece during the 2022–2023 competitive season [37]. The study protocol was approved by the Ethical Committee of the Faculty of Physiotherapy, University of Thessaly (542/3-5-2023), and complied with the Declaration of Helsinki. Data were collected from May to July 2023 using an electronic survey on the SurveyMonkey^®^ platform [37]. The study used the SurveyMonkey^®^ platform (https://www.surveymonkey.com/) and followed the Greek version of the Checklist for Reporting Results of Internet E-Surveys (CHERRIES) [38], recommended by the EQUATOR Network (www.equator-network.org) for web surveys.

### 2.2. Population/Participants

The sample comprised 222 male amateur footballers from the Greek Football Association amateur leagues (A, B, and C categories) over the season 2022–2023. Convenience and snowball sampling were used [39,40]. Eligible participants were male athletes over 18 actively competing in an amateur Greek football team; those under 18, in academies/development teams, or injured outside football were excluded.

### 2.3. Sample Calculation

Sample size was determined using the formula “n = (z^2^ × p × (1 − p))/e^2^”, where z = 1.96 (95% confidence), p is the estimated proportion, and e is the margin of error. Based on an estimated 406,655 athletes in Greece [41], a 50% injury prevalence [42], a 95% confidence level, and a 7% margin of error, a minimum of 196 participants was required.

### 2.4. Data Collection Procedures and Definitions

This study followed the international FIFA–UEFA consensus on football injury definitions and data collection [43,44,45], updated to address onset, recurrence, and illness registration [46]. Injuries were defined by time-loss and further classified by severity, location, and type [47,48,49]. Injury incidence was calculated as injuries per 1000 h of exposure (match, training, and total) over the 2022–2023 season, with 95% CIs using following the formulas of Knowles et al. (2006) [50]. Training exposure was estimated by multiplying weekly sessions by session duration over 32 weeks, while match exposure was based on the reported number of games played (×90 min).

### 2.5. Statistical Analysis

Raw data were transferred to Microsoft Excel 2021 (Microsoft Corp., Redmond, WA, USA), and statistical analyses were performed using SPSS version 25.0 (SPSS, Inc., Chicago, IL, USA) for Windows. Quantitative variables are presented as means ± standard deviations, and qualitative variables as percentages with 95% confidence intervals. The Kolmogorov–Smirnov test checked data normality; normally distributed variables are reported as the mean ± SD, and non-normally distributed variables as the median (IQR). All *p*-values were two-tailed at the 95% confidence level, with significance set at 0.05. Injuries are given as proportions (percent of total number of injuries) and incidence per 1000 h, with 95% confidence intervals. Effect sizes for Chi-square comparisons were also calculated [51]. The Phi Coefficient was applied for 2 × 2 tables, and Cramer’s V for larger tables. For comparisons between groups, one-way ANOVA followed by Bonferroni post hoc tests was used for parametric data, while Kruskal–Wallis tests with Dunn’s post hoc analyses were applied for non-parametric data.

## 3. Results

A total of 222 amateur football players aged 18 to 44 years (mean age: 25.25 ± 5.74 years) were included in the study and categorized into four groups based on their playing positions: defenders (DF; N = 63), forwards (FW; N = 61), midfielders (MF; N = 69), and goalkeepers (GK; N = 29). Demographic, anthropometric data and athletes’ profiles are summarized in Table 1. Statistically significant differences were observed in weight (*p* = 0.028) and height (*p* = 0.008), with GKs showing a higher weight than MFs (*p* = 0.025) and GKs being taller than MFs (*p* = 0.005) and FWs (*p* = 0.033). MFs had fewer training sessions per week compared to GKs (*p* = 0.036) and DFs (*p* = 0.033). Additionally, GKs had the lowest training exposure, showing significant differences with MFs (*p* = 0.035) and DFs (*p* = 0.003).

Table 2 provides an in-depth overview of the players’ general characteristics, organized by position. It includes details such as the amateur league category in which they compete, their leg dominance, the nature of physical effort in their work, and the type of playing surface. Additionally, the table offers insights into their participation in other forms of exercise, frequency of healthcare visits, passive recovery routines, number of training sessions, and the availability of a physical therapist or trainer during team practices.

### 3.1. Prevalence of Injuries

Table 3 provides an overview of the injury incidence, characteristics, and treatment among amateur football players during the 2022–2023 season. Forty-one DFs experienced at least one injury, resulting in an incidence proportion of 65.1% (95% CI: 53–77%). Similarly, 41 FWs reported injuries, with an incidence proportion of 67.2% (95% CI: 55–79%). MFs had a higher injury rate, with 52 players affected, corresponding to an incidence proportion of 75.4% (95% CI: 65–86%). Goalkeepers (GKs) showed the highest injury proportion, with 23 injured players reflecting 79.3% (95% CI: 65–94%). Despite these variations, there was no statistically significant association between player position and the likelihood of experiencing at least one injury during the 2022–2023 season (χ^2^ = 3.08, *p* = 0.379, Phi = 0.118). Over 80% of DFs, FWs, and MFs, along with approximately 70% of GKs, sought medical attention for their injuries, with physiotherapy being the most common treatment. Across all positions, treatment typically included rest and electric stimulation, with other therapies like massage and ice therapy also used. Most players, except GKs, were immediately removed from play following their injury. Additionally, a considerable number of players sustained recurrent injuries at the same site during the 2022–2023 season, with early recurrences being common. No statistically significant differences were found between playing positions.

### 3.2. Injury Rate

Aside from reporting at least one injury, players were also asked about any additional injuries they had. During the 2022–2023 season, a total of 220 injuries were recorded, resulting in an overall injury rate of 5.3 injuries/1000 h (95% CI: 4.61–6.01) [37]. DFs experienced 57 injuries over 12,647 h, with an overall incidence of 4.5 injuries/1000 h (95% CI: 3.34–5.68), MFs sustained the most injuries (73 injuries) over 12,057.5 h, resulting in an overall incidence of 5.7 injuries/1000 h (95% CI: 4.37–7.07). FWs reported 59 injuries across 10,964 h, with an overall incidence of 5.4 injuries/1000 h (95% CI: 4.01–6.75), and GKs accounted for 31 injuries over 5737.5 h, with an overall incidence of 5.4 injuries/1000 h (95% CI: 3.5–7.31). The distribution of the total numbers of injuries among amateur football players during the 2022–2023 season are presented in Supplementary Materials File S1.

### 3.3. Injuries in Matches vs. Training Sessions

**Figure 1:**
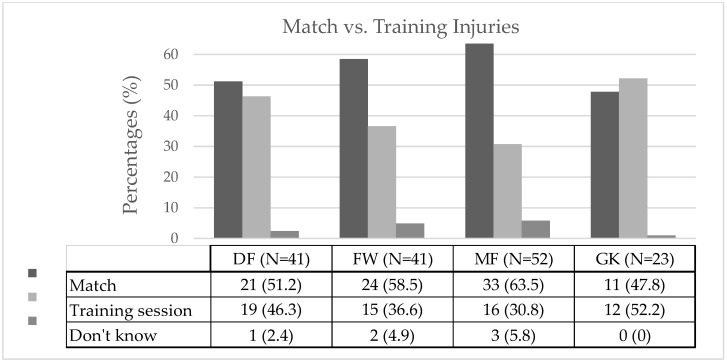
Injury proportion during matches and training sessions; DF = defender; FW = forward; MF = midfielder; GK = goalkeeper.

An analysis of the athletes’ most recent injuries revealed that the number of injuries did not significantly differ between games and training across the various playing positions (χ^2^ = 5.21, *p* = 0.517, Cramer’s V = 0.129) (Figure 1). However, the data suggest that DF, FW, and MF players are more prone to injuries during games compared to training. Specifically, DF players were 1.11-times more likely to be injured in games (21 injuries, 51.2% IR 1.66 injuries/1000 h, 95% CI: 0.95–2.37) compared to training (19 injuries, 46.3% IR 1.50 injuries/1000 h, 95% CI: 0.83–2.18). Similarly, FW players were 1.6-times more likely to sustain injuries during games (24 injuries, 58.5% IR 2.19 injuries/1000 h, 95% CI: 1.31–3.06) compared to training (15 injuries, 36.6% IR 1.37 injuries/1000 h, 95% CI: 1.31–3.06). MF players had the highest risk, being 2.06-times more likely to be injured during games (33 injuries, 63.5% IR 2.74 injuries/1000 h, 95% CI: 1.80–3.67) compared to training (16 injuries, 30.8% IR 1.33 injuries/1000 h, 95% CI: 0.68–1.98). In contrast, GK players were 1.09-times more likely to be injured during training (12 injuries, 52.2% IR 2.09 injuries/1000 h, 95% CI: 0.91–3.27) than in games (11 injuries, 47.8% IR 1.92 injuries/1000 h, 95% CI: 0.78–3.05).

### 3.4. Severity

**Figure 2:**
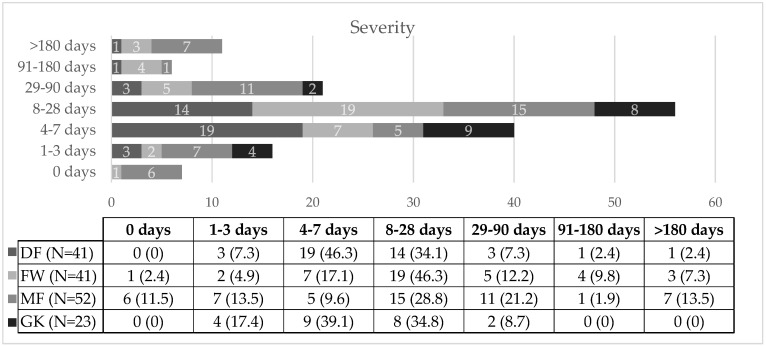
Severity of injuries. 0 days = slight injuries; 1–3 days = minimal injuries; 4–7 days = minor/mild injuries; 8–28 days = moderate injuries; 28 days and <8 weeks = major injuries; ≥8 weeks = severe injuries; DF = defender; FW = forward; MF = midfielder; GK = goalkeeper.

In terms of injury severity of the most recent injury (Figure 2), DFs experienced 46.3% minor injuries (19 injuries, IR 1.5 injuries/1000 h, 95% CI: 0.83–2.18) and 34.1% moderate injuries (14 injuries, IR 1.1 injuries/1000 h, 95% CI: 0.53–1.69). FWs had a higher proportion of moderate injuries at 46.3% (19 injuries, IR 1.73 injuries/1000 h, 95% CI: 0.95–2.51), while minor injuries comprised 17.1% (7 injuries, IR 0.64 injuries/1000 h, 95% CI: 0.17–1.11). MFs reported 28.8% moderate injuries (15 injuries, IR 1.24, injuries/1000 h, 95% CI: 0.61–1.87) and 21.2% major injuries (11 injuries, IR 0.91 injuries/1000 h, 95% CI: 0.37–1.45). GKs had 39.1% minor injuries (9 injuries, IR 1.57 injuries/1000 h, 95% CI: 0.54–2.59) and 34.8% moderate injuries (8 injuries, IR 1.39 injuries/1000 h, 95% CI: 0.43–2.36). Injury severity significantly varied by player position (χ^2^ = 44.42, *p* = 0.001, Cramer’s V = 0.307), with a notable difference between DFs and MFs (*p* = 0.013).

### 3.5. Injured Body Locations

**Figure 3:**
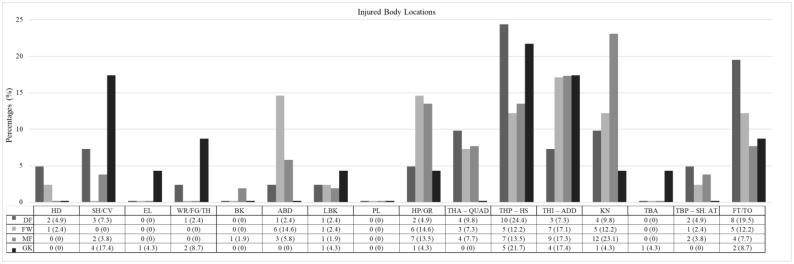
Injured body locations. DF = defender; FW = forward; MF = midfielder; GK = goalkeeper; HD = head; SH/CV = shoulder/clavicle; EL = elbow; WR/FG/TH = wrist/finger(s)/thumb; BK = back; ABD = abdomen; LBK = lower back; PL = pelvis; HP/GR = hip/groin; THA—QUAD = thigh (anterior)—quadriceps; THP—HS = thigh (posterior)—hamstrings; THI—ADD = thigh (inner)—adductors; KN = knee; TBA = tibia (anterior); TBP—SH. AT = tibia (posterior)—shank. Achilles tendon; FT/TO = foot/toe.

In this study, the most common injury locations varied by player position; however, there were no statistically significant differences between positions (χ^2^ = 57.68, *p* = 0.054, Cramer’s V = 0.350). DFs frequently sustained injuries to the posterior thigh (hamstrings) (10 injuries, 24.4%, IR 0.79 injuries/1000 h, 95% CI: 0.3–1.28), followed by foot/toe injuries (8 injuries, 19.5%, IR 0.63 injuries/1000 h, 95% CI: 0.19–1.07). FWs were primarily affected by injuries to the inner thigh (adductors) (7 injuries, 17.1%, IR 0.64 injuries/1000 h, 95% CI: 0.17–1.11), with additional significant injuries occurring in the hip/groin (6 injuries, 14.6%, IR 0.55 injuries/1000 h, 95% CI: 0.11–0.99) and abdomen (6 injuries, 14.6%, IR 0.55 injuries/1000 h, 95% CI: 0.11–0.99). MFs most commonly experienced knee injuries (12 injuries, 23.1%, IR 0.99 injuries/1000 h, 95% CI: 0.43–1.56), followed by inner thigh injuries (9 injuries, 17.3%, IR 0.75 injuries/1000 h, 95% CI: 0.26–1.23). GKs predominantly suffered from posterior thigh injuries (5 injuries, 21.7%, IR 0.87 injuries/1000 h, 95% CI: 0.11–1.64), along with inner thigh injuries (4 injuries, 17.4%, IR 0.7 injuries/1000 h, 95% CI: 0.01–1.38) and shoulder/clavicle injuries (4 injuries, 17.4%, IR 0.7 injuries/1000 h, 95% CI: 0.01–1.38) (Figure 3).

Lower-limb injuries were predominant across all positions, accounting for nearly 80% of injuries among DFs (33 injuries, 80.5%, IR 2.61, 95% CI: 1.72–3.5) and FWs (32 injuries, 80%, IR 2.92, 95% CI: 1.91–3.93). MFs experienced the highest proportion of lower-limb injuries at 86.5% (45 injuries, IR 3.73, 95% CI: 2.64–4.82). GKs, however, had a lower percentage of lower-limb injuries, at 63.6% (14 injuries, IR 2.44, 95% CI: 1.16–3.72) (Supplementary Materials File S2).

### 3.6. Injury Type

**Figure 4:**
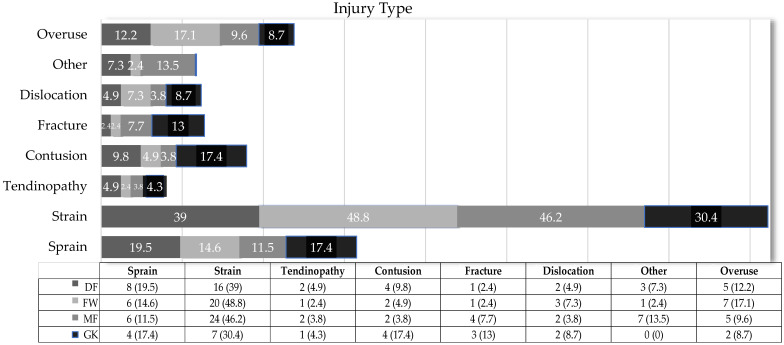
Injury type. DF = defender; FW = forward; MF = midfielder; GK = goalkeeper.

Regarding the type of injuries, strains were the most common across all player positions (Figure 4). DFs had the highest incidence of strains (16 injuries, 39%, IR 1.27 injuries/1000 h, 95% CI: 0.65–1.89), followed by sprains (8 injuries, 19.5%, IR 0.63 injuries/1000 h, 95% CI: 0.19–1.07). DFs were 2.02-times more likely to suffer strains than sprains. FWs were approximately 2.7-times more likely to suffer strains (20 injuries, 48.8%, IR 1.82 injuries/1000 h, 95% CI: 1.02–2.62) than overuse injuries (7 injuries, 17.1%, IR 0.64 injuries/1000 h, 95% CI: 0.17–1.11). While strains were the most common, overuse injuries occurred more frequently than sprains (6 injuries, 14.6%, IR 0.55 injuries/1000 h, 95% CI: 0.11–0.99). For MFs, strains were predominant (24 injuries, 46.2%, IR 1.99 injuries/1000 h, 95% CI: 1.19–2.79), with other types of injuries (7 injuries, 13.5%, IR 0.58 injuries/1000 h, 95% CI: 0.15–1.01) and sprains (6 injuries, 11.5%, IR 0.5 injuries/1000 h, 95% CI: 0.1–0.9) occurring less frequently. MFs were approximately 4-times more likely to suffer strains than sprains, and strains were 3.4-times more common than other types of injuries. Sprains were slightly less frequent compared to other injury types. GΚs primarily sustained muscle strains, accounting for 30.4% of injuries (7 injuries, IR 1.22 injuries per 1000 h, 95% CI: 0.32–2.12), followed by sprains at 17.4% (4 injuries, IR 0.7 injuries per 1000 h, 95% CI: 0.01–1.38) and contusions. The likelihood of goalkeepers experiencing strains was 1.74-times higher compared to sprains or contusions. There was no statistically significant difference between the injury type and player positions (χ^2^ = 19.51, *p* = 0.552, Cramer’s V = 0.204).

### 3.7. Injury Mechanisms

**Figure 5:**
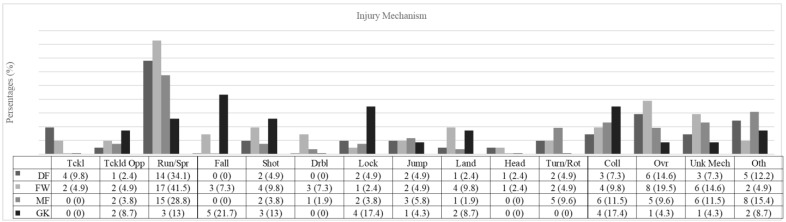
Injury mechanism. DF = defender; FW = forward; MF = midfielder; GK = goalkeeper; Tckl = tackling; Tckld Opp = tackled by opponents; Run/Spr = running/sprint; Fall = falling; Shot = shooting; Drbl = dribbling; Lock = locking; Jump = jumping; Land = landing; Head = heading; Turn/Rot = turning/rotation; Coll = collision; Ovr = overuse; Unk Mech = unknown mechanism; Oth = other.

Running or sprinting was the most common cause among DFs (14 injuries, 34.1%, IR 1.11 injuries/1000 h, 95% CI: 0.53–1.69), with overuse mechanisms being the second most frequent (6 injuries, 14.6%, IR 0.47 injuries/1000 h, 95% CI: 0.09–0.85). Similarly, FWs most often sustained injuries while running or sprinting (17 injuries, 41.5%, IR 1.55 injuries/1000 h, 95% CI: 0.81–2.29), followed by overuse (8 injuries, 19.5%, IR 0.46 injuries/1000 h, 95% CI: 0.22–1.24) and unknown mechanisms (6 injuries, 14.6%, IR 0.55, 95% CI: 0.11–0.99). For MFs, running or sprinting was also the leading injury mechanism (15 injuries, 28.8%, IR 1.24 injuries/1000 h, 95% CI: 0.61–1.87). In contrast, GKs most frequently sustained injuries due to falling (5 injuries, 21.7%, IR 0.87 injuries/1000 h, 95% CI: 0.11–1.64), followed by locking and collisions (4 injuries, 17.4%, IR 0.7 injuries/1000 h, 95% CI: 0.01–1.38) (Figure 5; Supplementary Materials File S2).

The analysis of injuries revealed varying distributions of non-contact and contact injuries among different player positions. For DFs, non-contact injuries comprised 31.7% of all injuries (13 injuries, IR 1.03 injuries/1000 h, 95%CI: 0.47–1.59), while contact injuries accounted for 26.8% (11 injuries, IR 0.87 injuries/1000 h, 95% CI: 0.36–1.38). FWs showed a similar pattern, with non-contact injuries making up 31.7% (23 injuries, IR 2.1 injuries/1000 h, 95% CI: 0.22–1.24) and contact injuries 26.8% (13 injuries, IR 1.19 injuries/1000 h, 95% CI: 0.54–1.83). Among MFs, non-contact injuries were more prevalent at 32.6% (17 injuries, IR 1.41 injuries/1000 h, 95% CI: 0.74–2.08) compared to 19.1% for contact injuries (10 injuries, IR 0.83 injuries/1000 h, 95% CI: 0.32–1.34). In contrast, GKs exhibited a higher incidence of contact injuries, which constituted 65.2% of their total injuries (15 injuries, IR 2.61 injuries/1000 h, 95% CI: 1.29–3.94), while non-contact injuries were 30.3% (7 injuries, IR 1.22, 95% CI: 0.32–2.12) (Supplementary Materials File S2).

### 3.8. Characteristics of Reported Additional Injuries

**Figure 6:**
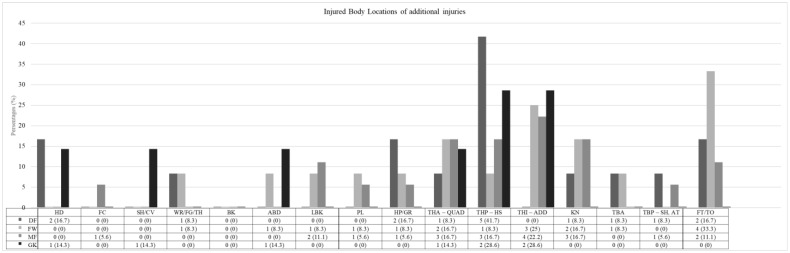
Injured body locations of additional injuries. DF = defender; FW = forward; MF = midfielder; GK = goalkeeper; HD = head; FC = Face; SH/CV = shoulder/clavicle; EL = elbow; WR/FG/TH = wrist/finger(s)/thumb; BK = back; ABD = abdomen; LBK = lower back; PL = pelvis; HP/GR = hip/groin; THA—QUAD = thigh (anterior)—quadriceps; THP—HS = thigh (posterior)—hamstrings; THI—ADD = thigh (inner)—adductors; KN = knee; TBA = tibia (anterior); TBP—SH. AT = tibia (posterior)—shank. Achilles tendon; FT/TO = foot/toe.

The analysis did not show a statistically significant effect of playing position on the occurrence of additional injuries (χ^2^ = 0.431, *p* = 0.934, Cramer’s V = 0.052). The majority of athletes in each position did not experience additional injuries. Among positions, MFs had the highest rate of additional injuries (34.6%), while DFs and FWs had the lowest rate (29.3%). Most of these injuries occurred during matches for all positions, except for FWs, who had a higher incidence of injuries during training sessions (50%). Table 4 provides data on additional injuries sustained by players during the 2022–2023 season, including the occurrence of injuries, their timing (match or training), removal from play, time off football, healthcare visits, treatments followed, and types of physiotherapy interventions across different player positions. Figure 6 presents the injury distribution of the additional injuries athletes reported.

DFs exhibited a higher frequency of injuries to the posterior thigh (41.7%) and the hip/groin area (16.7%) (Figure 6). FWs experienced more injuries to the foot/toe (33.3%) and the anterior thigh and knee (16.7% each). MFs showed a broader variety of injuries, with the most common being the adductors (22.2%) followed by the anterior and posterior thigh (16.7% each). GKs most frequently sustained injuries to the posterior and inner thigh (28.6% each), as well as to the head (14.3%). Strains were the most common additional injury across all positions, with MFs reporting nine injuries (50.0%, IR 0.75, 95% CI: 0.26–1.23), DFs seven injuries (58.3%, IR 0.55, 95% CI: 0.14–0.96), FWs five injuries (41.7%, IR 0.46, CI: 0.06–0.86), and GKs four injuries (57.1%, IR 0.70, 95% CI: 0.01–1.38). Overuse injuries were mainly reported among MFs (4 injuries, 22.2%, IR 0.33, 95% CI: 0.01–0.66), with minimal occurrences in other positions. The characteristics of the reported additional injuries with corresponding injury rates are presented in Supplementary Materials File S3.

### 3.9. Prevention

**Figure 7:**
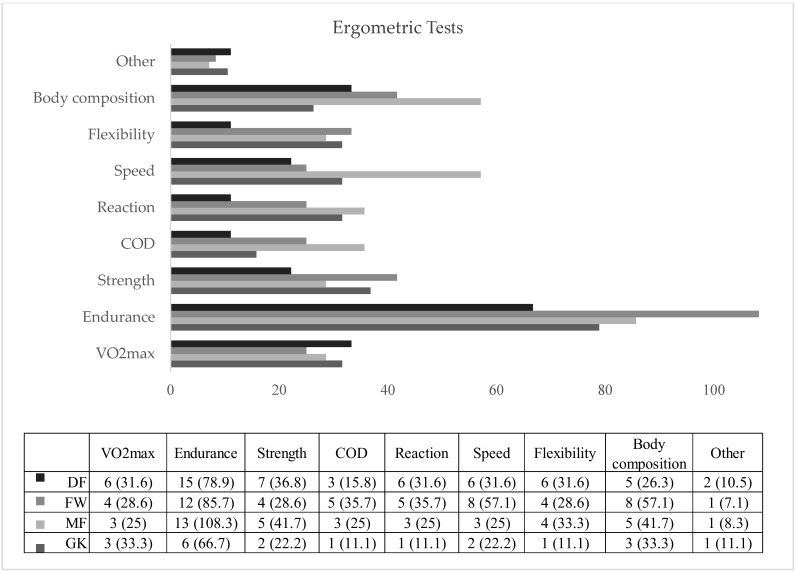
Distribution of ergometric tests performed by playing position; DF = defender; FW = forward; MF = midfielder; GK = goalkeeper.

Figure 7 provides data on the participation of players in ergometric tests during the 2022–2023 season, categorized by playing position. Overall, participation in such tests was low across all groups, though DFs and GKs demonstrated relatively higher engagement (30.2% and 31%, respectively), compared to FWs and MFs. Notably, MFs exhibited the highest participation in endurance assessments (108.3%), but the lowest in change-of-direction (COD) tests (25%). FWs had the highest involvement in speed testing (57.1%), while goalkeepers showed the lowest participation in strength and speed assessments. DFs demonstrated a more balanced distribution across test types, with greater emphasis on endurance and speed evaluations. These differences may reflect positional demands or training emphasis within amateur teams.

Table 5 illustrates players’ awareness and application of specific injury prevention exercises by playing position. Awareness levels were generally high across all positions, with goalkeepers showing the highest awareness—96.6% knew the Nordic hamstring and front plank exercises, and 100% were familiar with the Copenhagen adductor exercise. DFs and MFs also demonstrated strong awareness, particularly for core and groin-focused exercises such as the front plank and Copenhagen adductor. FWs had slightly lower levels of knowledge, though still reported good familiarity with key prevention exercises.

In terms of implementation, GKs and MFs demonstrated the highest overall engagement in injury prevention exercises. GKs most frequently reported using these exercises both in-season and pre-season (51.7%), followed by substantial use in the pre-season alone (31%). Similarly, MFs predominantly used them during both periods (42%) or exclusively in the pre-season (34.8%). DFs reported a more even distribution, with 49.2% performing injury prevention in-season and 30.2% across both periods. FWs were more variable, but a considerable portion reported regular use both in-season and pre-season (42.6%).

## 4. Discussion

This study investigated the epidemiology of injuries in amateur football players in Greece, focusing on the role of playing position in injury incidence, characteristics, severity, location, and mechanisms. The findings confirm that amateur football players, regardless of position, are at a high risk of injury, with positional role influencing not only injury type but also severity and mechanism.

Previous research examining the influence of playing position on injury risk in male football has produced inconsistent results. Several studies report a higher prevalence of injuries among FWs [27,31,34,52,53,54,55,56,57,58,59,60,61,62,63,64,65,66], while others indicate that MFs are more prone to injuries [26,56,57,58], and a few emphasize DFs as the most vulnerable group [59,60,61,62]. Some work, such as Mallo (2012) [66], highlighted that both FWs and central DFs share a similar high risk of injury, whereas other investigations noted that GKs are either the most frequently injured players [63] or, conversely, at the lowest risk overall [54,55,64]. Moreover, Aoki et al. (2012) [64] pointed out that, despite their generally lower injury rates, goalkeepers are particularly susceptible to head, face, and upper-extremity injuries because of the unique demands of their role [53,65,67]. In our study of amateur Greek footballers, we also found no statistically significant association between playing position and injury risk. Although GKs demonstrated the highest injury prevalence (79.3%), followed by MFs (75.4%), FWs (67.2%), and DFs (65.1%), these differences did not reach statistical significance. When exposure was considered, injury incidence was highest in MFs (5.7 injuries/1000 h), followed by FWs and GKs (both 5.4 injuries/1000 h) and DFs (4.5 injuries/1000 h). These results align with studies that reported no meaningful positional differences [26,52,55,60,62,63,65,66,68,69] and contrast with others that have identified a clear association between position and injury occurrence [27,54,58,59,64]. The lack of consistent patterns across studies likely reflects differences in study populations, competitive levels, and injury definitions. Overall, our findings suggest that in amateur male football, injury risk may not be strongly influenced by playing position, supporting the need for prevention strategies that target all players irrespective of field role.

This discrepancy between injury prevalence and incidence observed in our study highlights the importance of distinguishing between these two complementary measures. Prevalence reflects the proportion of players who sustained at least one injury during the season, without taking into account the total exposure time or number of injuries per individual. In contrast, injury incidence (injuries per 1000 h of exposure) adjusts for exposure and provides a more accurate estimate of the frequency of injuries during play and training. Consequently, GKs in our cohort showed the highest prevalence, meaning that a larger proportion of goalkeepers experienced at least one injury, whereas MFs exhibited the highest incidence because of their greater cumulative exposure and the high physical demands of their position. Similar patterns, where prevalence and incidence identify different at-risk groups, have been described in previous injury surveillance work (e.g., [33,40,44]). These differences emphasize that both metrics should be considered when interpreting injury risk and designing prevention strategies in football.

A possible explanation for the absence of significant differences in injury incidence between playing positions in our study may lie in the evolving nature of modern football, where positional roles are increasingly dynamic [5,70,71]. Players frequently alternate between offensive and defensive responsibilities throughout a match, regardless of their primary role. For example, fullbacks often function as attackers during ball possession phases but revert to defensive duties when possession is lost. Similarly, FWs and DFs are required to perform both attacking and defending tasks, increasing their overall physical load and exposure to high-intensity actions such as sprints, accelerations, and frequent directional changes, all of which elevate the risk of intrinsic injuries like muscle strains [23,72]. Regarding GKs, the relatively small sample size of players in this position could limit the statistical power needed to detect significant differences in injury prevalence or rates compared to field players.

### 4.1. Injuries During Matches vs. Training

In the present study, no statistically significant differences were found in the number of injuries between matches and training sessions across different playing positions. Nevertheless, certain trends emerged: players, irrespective of position, were more likely to sustain injuries during matches than during training. MFs exhibited the highest relative risk, being 2.06-times more likely to be injured during matches than training, followed by FWs (1.6 times). This finding aligns partially with existing literature, which presents mixed results regarding the relationship between playing position and injury risk in matches versus training. While some studies focus exclusively on match injuries [3,27,54,55,61,69,73], reporting higher injury rates in FWs and DFs during match play [61,69,73], others examining both match and training injuries [26,59,62,74,75] report no significant influence of playing position on injury occurrence. The current results, though not statistically significant, highlight that amateur players—similar to professionals—are generally at higher injury risk during competitive play.

### 4.2. Injured Body Locations

Lower-limb injuries predominated across all positions in the current study, confirming previous reports in both professional and amateur football [6,26,33,34,56,76]. This distribution reflects the physical demands of football, which primarily involve lower-limb actions such as sprinting, kicking, and directional changes [6,26,76]. While no statistically significant positional differences were observed regarding injury location, certain trends were identified. DFs more frequently sustained posterior thigh (hamstring) and foot/toe injuries, likely due to frequent tackling, sudden decelerations, and rapid directional changes. FWs exhibited a higher prevalence of inner thigh (adductor), hip/groin, and abdominal injuries, possibly reflecting the high demands of sprinting, shooting, and explosive movements. MFs, who typically cover the greatest distances and perform constant multidirectional movements, showed a higher incidence of knee injuries, followed by adductor injuries. GKs predominantly sustained posterior thigh, inner thigh (adductor), and shoulder/clavicle injuries, which may be attributed to repetitive jumping, diving, and falls inherent to their specialized role.

### 4.3. Upper-Limb Injuries

Upper-limb injuries are generally uncommon in football, as the sport predominantly involves lower-limb activity. When such injuries do occur, they typically involve short periods of morbidity and removal from play [77]. However, GKs represent a notable exception, sustaining upper-limb injuries at significantly higher rates than outfield players—up to five-times more frequently, as reported in previous studies [33,34,64]. These injuries often result from repetitive hand use, ball interceptions, falls, and collisions, commonly affecting the wrist, hand, and shoulder joints, with an average absence of approximately three weeks [34]. In the present study, GKs exhibited a higher, though not statistically significant, tendency for upper-limb injuries compared to field players, consistent with the literature. Interestingly, DFs also showed a relatively higher occurrence of upper-limb injuries than other outfield positions, likely related to falls during defensive actions and aerial duels—a finding supported by Hunt & Fulford (1990) [33]. Additionally, although head and neck injuries were infrequent, they were reported mainly in DFs and FWs, possibly due to their involvement in physical contact and aerial challenges, as similarly noted by Forsythe et al. (2022) [56]. Overall, these findings highlight the distinct injury risk profile of GKs regarding upper-limb injuries, while also suggesting that DFs may face a greater risk than previously assumed.

### 4.4. Injury Type

The present study found that muscle injuries were the most common type of injury across all playing positions among amateur football players in Greece, with MFs being the most affected. Specifically, MFs were approximately 3.4-times more likely to sustain muscle strains compared to other types of injuries, although no statistically significant differences in injury type were observed between positions. These findings are in line with previous research, which consistently identifies muscle injuries as the predominant injury type in football. Onaka et al. (2017) [34] reported that muscle injuries, particularly affecting the back muscles, were more frequent among advanced MFs, while hamstring strains were common among wingers and FWs. Conversely, Arliani et al. (2011) [78] found no significant association between playing position and injury type, although muscle strains were the most frequent injury across nearly all positions except GKs. Similarly, Chahla et al. (2018) [52] described varied injury profiles across positions, noting a higher occurrence of muscular injuries in FWs and a broader variety of injury types—including contusions and tendon injuries—among DFs. Reis et al. (2015) [57] and Carling et al. (2010) [27] further support the role of playing position as a contributing factor in injury type distribution, with wingbacks and central forwards showing increased susceptibility to muscle and tendon injuries. More recently, Thema et al. (2025) [79] confirmed that midfielders exhibited the highest frequency of soft-tissue injuries among sub-elite male players, reinforcing the positional trend observed in the present study. Overall, the predominance of muscle strains in this study, particularly among MFs, aligns with the majority of existing literature and underscores the importance of position-specific injury prevention strategies in amateur football.

The positional distribution of muscle injuries observed in this study likely reflects the differing physical and biomechanical demands associated with each role on the field. MFs, who demonstrated the highest proportion of muscle strains, typically act as the link between defensive and attacking phases of play. This role requires constant movement, frequent directional changes, and rapid transitions, all of which increase stress on muscles, tendons, and joints, potentially predisposing these players to soft-tissue injuries [79,80]. Similarly, FWs—who also exhibited a high incidence of muscle injuries—engage in repeated accelerations, decelerations, and sprinting actions, movements known to overload muscular structures [9,23]. These patterns are consistent with prior studies noting that FWs and wing players perform the highest number of sprints per match, due to their dual offensive and defensive responsibilities during play [9,23]. In contrast, DFs in the present study sustained a relatively broader variety of injuries, possibly due to their involvement in tackling and aerial duels rather than continuous high-speed running [23]. The lower severity of injuries in DFs could also be linked to their generally lower sprinting demands during matches [23]. Overall, the injury profiles identified in this study appear to be closely associated with the unique physical and tactical demands placed on players according to their positional role.

### 4.5. Injury Severity

Injury severity in relation to playing position remains an underexplored area in football injury research. While numerous studies have focused on injury incidence per position, few have assessed whether the severity of injuries varies depending on the player’s role on the field. To our knowledge, only a limited number of studies [55,57,60,65] have examined this aspect, often as a secondary outcome. Most of these studies prioritized identifying which positions are more frequently injured, overlooking how severe those injuries tend to be. This represents an important gap in the literature, as understanding injury severity is essential for developing targeted, position-specific prevention and rehabilitation strategies. Considering the differing physical demands, movement patterns, and exposure to contact situations across playing positions, it is plausible that not only injury incidence but also injury severity may be influenced by positional role.

The existing literature presents mixed findings. Paulo (2017) [55] and Dauty et al. (2011) [60] found no significant differences in injury severity between positions, while Reis et al. (2015) [57] reported a significant association, with DFs sustaining less severe injuries and FWs more moderate to severe injuries. Le Gall et al. (2006) [65] identified GKs as sustaining the highest percentage of moderate and major injuries, though without statistical significance.

In contrast, the present study demonstrated that injury severity varied significantly according to playing position (*p* = 0.001), contributing novel data to this underexplored area. DFs and GKs predominantly sustained minor and moderate injuries, while FWs exhibited a higher proportion of moderate injuries. MFs experienced the highest proportion of major injuries, suggesting that their role imposes greater physical demands and increases the risk of severe injuries. The lower injury severity in DFs may be explained by their reduced sprinting and overall physical load [23,57]), while the greater severity observed in FWs and MFs likely reflects their exposure to repeated high-intensity efforts, accelerations, and multidirectional movements. MFs’ constant involvement in both offensive and defensive phases, combined with high running volumes, may explain their susceptibility to more severe injuries. Collectively, these findings emphasize the importance of including injury severity in epidemiological analyses, as positional role appears to influence not only injury incidence but also the severity of injuries sustained. These findings partially align with those of Reis et al. [57], who linked the lower severity of injuries in defenders to their reduced sprinting demands and lower cumulative physical loads during matches. Similarly, the higher severity observed in forwards and midfielders in the present study may reflect the more intense physical demands and high-volume, high-intensity movements inherent in their roles. Forwards are often required to perform repeated maximal sprints and accelerations, while midfielders combine continuous running with frequent changes of direction and involvement in both attacking and defensive phases. These biomechanical and physiological demands may explain their greater susceptibility to more severe injuries.

### 4.6. Injury Mechanisms

Injury mechanism analysis revealed that most injuries among DFs, FWs, and MFs were non-contact in nature, predominantly resulting from running or sprinting actions. This reflects the high physical demands and repeated high-intensity efforts required by these positions during matches. Conversely, GKs sustained most of their injuries through contact mechanisms, such as falls, collisions, and physical contact with other players—an observation consistent with the specific biomechanical and functional demands of their role. The literature examining injury mechanisms in relation to playing position is scarce. Onaka et al. (2017) [34], using a different classification system, reported that trauma represented the main cause of sports injuries across all positions. However, their findings did not differentiate between contact and non-contact mechanisms per positional role. The current results underline the importance of considering injury mechanisms alongside injury type and severity, as understanding how injuries occur in relation to positional demands can inform the design of targeted preventive interventions.

### 4.7. Injury Prevention Awareness and Practices

The present study found a generally high level of awareness regarding key injury prevention exercises among amateur football players across all positions, with GKs demonstrating the highest awareness rates. Notably, over 95% of GKs were familiar with widely recommended exercises such as the Nordic hamstring, front plank, and Copenhagen adductor exercises. DFs and MFs also exhibited strong awareness, particularly for core and groin-focused routines. Although FWs showed slightly lower levels of familiarity, their awareness remained relatively high overall.

In terms of practical application, the data revealed variation in when players incorporate injury prevention exercises into their routines. GKs and MFs were the most consistent users, with a significant proportion reporting the use of these exercises during both the pre-season and in-season periods. DFs showed a similar trend, although their use was more heavily concentrated during the season. FWs were more variable in timing, with some relying primarily on pre-season routines.

These findings align with previous research suggesting that awareness of injury prevention strategies does not always translate into consistent practice, particularly in amateur settings where structured support and supervision may be limited [81,82]. The variability observed between positions could be influenced by differences in coaching emphasis, access to physiotherapy guidance, or perceived injury risk. For instance, GKs may have more individualized training and supervision, which can facilitate both greater awareness and more structured injury prevention routines [82].

Furthermore, the high awareness of exercises such as the Copenhagen adductor and Nordic hamstring—both of which have been shown to reduce muscle injury risk [83,84,85,86]—indicates positive dissemination of evidence-based strategies even at the amateur level. However, inconsistent application across time periods and positions suggests that additional education and implementation support may be needed to ensure injury prevention programs are not only known but also consistently followed throughout the season [87,88].

Given the generally high awareness but variable implementation of injury prevention exercises observed in this study, there is a clear need for more structured and consistent integration of such programs into amateur football training routines. Coaches, fitness trainers, and club staff should be educated on the value of evidence-based prevention strategies and encouraged to include them as a standard component of both pre-season and in-season practice [87,88]. Position-specific approaches may also be beneficial, as physical demands vary among roles. For instance, MFs—who experience high workloads—may benefit from programs emphasizing eccentric strength and endurance, while GKs may require routines focused on upper body control and joint stability [84,85]. Additionally, increased use of ergometric testing could help to identify individual physical deficiencies and allow for more tailored prevention strategies [82,89]. Since DFs and GKs showed relatively higher participation in ergometric assessments, similar practices could be promoted among other positions to enhance injury risk profiling and program targeting [90].

### 4.8. Limitations

The present study has several limitations that should be considered when interpreting its findings. Its retrospective design, based on self-reported questionnaire data, may have introduced recall bias and reduced accuracy, as players might lack detailed knowledge of their injuries, unlike assessments performed by qualified medical professionals. Because exposure hours were estimated from self-reported numbers and durations of training sessions and matches, small inaccuracies in recall may have influenced the calculated incidence rates. No sensitivity analysis was performed to examine the robustness of these estimates, and the results should therefore be interpreted with caution. Moreover, the retrospective nature of the study restricts the ability to accurately estimate injury incidence, since exposure times were derived from participants’ recollections. The absence of medical examinations potentially led to underreporting or misclassification of injuries compared to prospective studies with clinical diagnoses. Data were collected at the end of a single competitive season, limiting the ability to analyze injury trends over longer periods or across multiple seasons.

Further limitations relate to the data on injury prevention practices. The self-reported nature of awareness and exercise use may be subject to recall or social desirability bias, particularly if players overstated their engagement. Moreover, while the study documented whether players used specific exercises and during which periods, it did not assess frequency, intensity, or quality of execution—factors that are critical to the actual effectiveness of prevention strategies. Although data were collected on prevention exercises and recovery strategies, no analysis was conducted to examine their potential impact on injury outcomes. This was due to the retrospective design, self-reported data, and the relatively small number of players adopting specific methods (e.g., Nordic hamstring exercise). Additionally, the study did not distinguish whether implementation was athlete-led or guided by coaches or staff, which may influence adherence. The study did not control for several potential confounders, such as fitness level, detailed injury history, medical care access, type of field, playing style, and match load. These factors could influence injury risk and may partly explain differences between playing positions. Moreover, the analyses were descriptive and did not employ multivariable regression models to adjust for potential confounders, which may limit the interpretation of certain associations.

Beyond methodological considerations, limitations also arise when comparing the present findings to the existing literature. Different studies adopt inconsistent definitions of playing positions, making direct comparison of position-specific injury patterns challenging. An additional limitation of the present work is that the analysis classified players only into four broad positional categories (GKs, DFs, MFs, FWs). However, previous research has demonstrated substantial differences in duties and physical demands between players occupying wide versus central roles within the same line (e.g., central DFs versus full-backs, or central MFs versus wide MFs) [70]. Furthermore, other authors emphasize that there may even be meaningful differences in roles and demands among players who share the same nominal position, such as between different types of central MFs [91,92]. Furthermore, many studies evaluate only match-related injuries, neglecting injuries sustained during training, which constitute a substantial proportion of football injuries. Some studies fail to report whether their incidence rates are based on total (match and training) exposure time or omit exposure time definitions altogether. A further complication is the inconsistency in reported outcomes: some studies focus on injury prevalence, while others report injury incidence rates, which reflect injury risk per hours of play and are therefore more suitable for risk comparisons. Additionally, several previous studies investigate youth populations, where biological maturation affects injury patterns, limiting their comparability with adult amateur players. Finally, due to the scarcity of research involving amateur footballers, comparisons in this study often relied on professional-level data, despite the clear differences in training conditions, match load, and medical support between these two populations. Variations in injury classification systems and failure to report position-specific incidence rates, as opposed to general injury proportions, further hinder direct comparison with existing evidence.

The use of online convenience and snowball sampling methods may have introduced selection bias, restricting the representativeness of the sample and limiting the generalizability of the findings to all amateur footballers in Greece. Furthermore, the sample size calculation employed a 7% margin of error rather than the conventional 5%, reflecting practical constraints, and although the required minimum was exceeded, the estimates should still be interpreted with caution. The study did not include comprehensive socioeconomic data, nor did it prospectively track lifestyle factors such as sleep or recovery practices. Although survey questions on healthcare access, recovery, and rehabilitation provided a general descriptive overview, these aspects should be examined in more detail in future studies.

### 4.9. Future Implications

Building on these findings, amateur football stakeholders—including coaches, club staff, and governing bodies—should prioritize the integration of structured, evidence-based injury prevention programs throughout the season. Exercises such as the Nordic hamstring and Copenhagen adductor should be consistently applied and adapted to the specific demands of each playing position. Emphasis should also be placed on player education and coach-led implementation, particularly in settings with limited access to medical or performance professionals.

National and regional football associations could support this effort through standardized guidelines, educational initiatives, and greater use of accessible technologies—such as mobile apps or wearable devices—to monitor training load and adherence in real time. From a research perspective, future studies should employ prospective, longitudinal designs with objective injury surveillance and standardized definitions to improve data accuracy and comparability. Expanding this research across diverse amateur populations will help to tailor interventions to the unique needs of grassroots football and enhance player safety and performance at all levels.

## 5. Conclusions

While injury prevalence and injury rates varied across positions—with GKs exhibiting the highest prevalence and MFs the highest rate—no statistically significant association was found between playing position and overall injury risk among male amateur football players in Greece. Muscle injuries, particularly in the lower limbs, were the most common across all positions, with MFs and FWs showing a higher tendency toward severe and recurrent injuries. Although players demonstrated high awareness of evidence-based prevention exercises, such as the Nordic hamstring and Copenhagen adductor, their implementation varied across positions and training phases. These findings suggest that injury risk in amateur football is influenced more by overall physical demands and training exposure than by fixed positional roles. Position-sensitive but broadly applied prevention strategies, implemented consistently throughout the season, may be more effective in reducing injury burden. Future research using prospective methods and objective injury tracking is recommended to validate these trends and inform more targeted interventions.

## Figures and Tables

**Table 1:** Demographic, anthropometric data and athletes’ profiles per playing position.

Ν = 222	Defenders (DF) (N = 63)			Forwards (FW) (N = 61)			Midfielders (MF) (N = 69)			Goalkeepers (GK) (N = 29)			Total (Ν = 222)		
	Range (Min–Max)	Mean	Stdv	Range (Min–Max)	Mean	Stdv	Range (Min–Max)	Mean	Stdv	Range (Min–Max)	Mean	Stdv	Range (Min–Max)	Mean	Stdv
Age (years)	26 (18–44)	25.6	6.48	22 (18–40)	25.26	5.74	19 (18–37)	25.41	5.28	17 (18–35)	24.07	5.21	26 (18–44)	25.25	5.74
Height (cm) ^‡^	28 (168–196)	179.35	6.82	29 (164–193)	178.33	5.89	23 (167–190)	177.67	5.54	15 (174–189)	181.76 ^£⸣^	3.92	32 (164–196)	178.86	5.96
Weight (kg) ^‡^	39 (56–95)	76.75	8.54	49 (56–105)	75.1	9.3	35 (59–94)	74.59 *	7.53	46 (64–110)	80.02	9.71	54 (56–110)	76.05	8.75
BMI (kg/m^2^)	10.7 (18.71–29.41)	23.83	2.05	9.67 (18.52–28.19)	23.57	2.26	9.62 (19.44–29.07)	23.63	2.1	11.47 (19.32–30.79)	24.19	2.48	12.28 (18.52–30.79)	23.74	2.18
Exp (years)	25 (1–26)	8.6	6.26	19 (1–20)	8.4	5.86	21 (1–22)	8.57	5.71	27 (1–28)	9.25	7.21	49 (1–50)	8.93	6.88
Sessions/wk (days) ^‡^	4 (2–6)	3.44	1.01	4 (2–6)	3.9	1.32	5 (1–6)	3.23 ^#§^	1.14	4 (2–6)	3.76	1.06	5 (1–6)	3.53	1.13
Duration (min)	90 (30–120)	84.29	16.48	90 (30–120)	81.39	16.76	90 (30–120)	77.39	13.76	75 (45–120)	81.21	16.29	90 (30–120)	80.95	15.86
Rest/wk (days)	4 (1–5)	2.36	0.95	5 (0–5)	2.41	1.18	5 (0–5)	2.51	1.09	3 (1–4)	2.29	0.91	5 (0–5)	2.39	1.01
TrE/1000 h ^#^	10,736			9152			9272 ^†⁑^			4920			34,080		
ME/1000 h	1911			1812			2785.5			817.5			7326		
ToE/1000 h	12,647			10,964			12,057.5			5737.5			41,406		

BMI = body mass index; Stdv = standard deviation; Min = minimum; Max = maximum; TrE/1000 h = training exposure/1000 h; ME/1000 h = match exposure/1000 h; ToE/1000 h = total exposure/1000 h; Exp = experience; Sessions/wk = training sessions/week; Duration = training duration; Rest/wk = rest days/week; * *p* = 0.025 vs. GK; ^⁑^ *p* = 0.035 vs. GK; ^†^ *p* = 0.003 vs. DF; ^‡^ *p* < 0.05 among groups; ^#^ *p* = 0.036 vs. GK; ^§^ *p* = 0.033 vs. DF; ^£^ *p* = 0.005 vs. MF; ⸣ *p* = 0.033 vs. FW.

**Table 2:** General characteristics and playing profiles of amateur football players in Greece. The values in the table are presented as numbers (percentages).

N = 222	DF (N = 63)	FW (N = 61)	MF (N = 69)	GK (N = 29)
**Amateur league category**				
A League	41 (65.1)	40 (65.6)	45 (65.2)	17 (58.6)
B League	18 (28.6)	16 (26.2)	15 (21.7)	8 (27.6)
C League	4 (6.3)	5 (8.2)	9 (13)	4 (13.8)
**Leg dominance**				
Left	12 (19)	14 (23)	16 (23.2)	4 (13.8)
Right	45 (71.4)	45 (73.8)	48 (69.6)	22 (75.9)
Both	6 (9.5)	2 (3.3)	5 (7.2)	3 (10.3)
**Type of physical effort that work involves**				
Sedentary	20 (31.7)	22 (36.1)	29 (42)	10 (34.5)
Standing and walking	20 (31.7)	17 (27.9)	20 (29)	7 (24.1)
Standing, walking, and lifting	15 (23.8)	16 (26.2)	15 (21.7)	7 (24.1)
Other	8 (12.7)	6 (9.8)	5 (7.2)	5 (17.2)
**Type of surface**				
Synthetic/artificial turf	31 (49.2)	24 (39.3)	27 (39.1)	12 (41.4)
Νatural grass	32 (50.8)	35 (57.4)	40 (58)	16 (55.2)
Dirt surface	0 (0)	2 (3.3)	1 (1.4)	1 (3.4)
Don’t know	0 (0)	0 (0)	1 (1.4)	0 (0)
**Engagement in any other form of exercise beyond football**				
Yes	34 (54)	37 (60.7)	35 (50.7)	18 (62.1)
No	29 (46)	24 (39.3)	34 (49.3)	11 (37.9)
**Regularly visit a healthcare professional**				
Yes	21 (33.3)	22 (36.1)	18 (26.1)	9 (31)
No	42 (66.7)	39 (63.9)	51 (73.9)	20 (69)
Healthcare professional specialty *				
Physiotherapist	13 (61.9)	12 (54.5)	10 (55.6)	5 (55.6)
Nutritionist	0 (0)	2 (9.1)	0 (0)	1 (11.1)
Doctor	5 (23.8)	6 (27.3)	6 (33.3)	3 (33.3)
Other	3 (14.3)	2 (9)	2 (11.1)	0 (0)
Total	21 (100)	22 (100)	18 (100)	9 (100)
**Passive recovery**				
Yes	26 (41.3)	17 (27.9)	17 (24.6)	17 (24.6)
No	37 (58.7)	44 (72.1)	52 (75.4)	52 (75.4)
**Number of training sessions in the 2022–2023 season**				
Number	27 (42.9)	22 (36.1)	26 (37.7)	12 (41.4)
Don’t know	36 (57.1)	39 (63.9)	43 (62.3)	17 (58.6)
**Physical therapist presence at the team’s training sessions**				
Yes	13 (20.6)	16 (26.2)	9 (13)	4 (13.8)
No	50 (79.4)	45 (73.8)	60 (87)	25 (86.2)
**Trainer present at the team’s training sessions**				
Yes	32 (50.8)	32 (52.5)	35 (50.7)	13 (44.8)
No	31 (49.2)	29 (47.5)	34 (49.3)	16 (55.2)

DF = defender; FW = forward; MF = midfielder; GK = goalkeeper. * Participants could choose up to two answers.

**Table 3:** Injury incidence, characteristics and treatment among amateur football players during the 2022–2023 football season. The values in the table are presented as numbers (percentages).

N = 222	DF	FW	MF	GK
**At least one injury in the football season 2022–2023**				
Yes	41 (65.1)	41 (67.2)	52 (75.4)	23 (79.3)
No	22 (34.9)	20 (32.8)	17 (24.6)	6 (20.7)
**Removal from play**				
Immediately	22 (53.7)	22 (53.7)	25 (48.1)	5 (21.7)
Later	13 (31.7)	10 (24.4)	12 (23.1)	6 (26.1)
Not at all	6 (14.6)	9 (22)	15 (28.8)	12 (52.2)
**Visit a healthcare professional—rehabilitation specialist**				
Yes	37 (90.2)	36 (87.8)	43 (82.7)	16 (69.6)
No	4 (9.8)	5 (12.2)	9 (17.3)	7 (30.4)
Kind of healthcare professional *				
Doctor	10 (24.4)	11 (26.8)	11 (21.2)	6 (26.1)
Physiotherapist	24 (58.5)	25 (61)	29 (55.8)	11 (47.8)
Other	4 (9.8)	0 (0)	3 (5.8)	1 (4.3)
Treatment followed *				
Physiotherapy	32 (86.5)	30 (83.3)	38 (88.4)	11 (68.8)
Rest	14 (37.8)	17 (47.2)	12 (27.9)	7 (43.8)
Medication	6 (16.2)	3 (8.3)	6 (14)	2 (12.5)
Injections	2 (5.4)	2 (5.6)	0 (0)	0 (0)
Surgery	1 (2.7)	2 (5.6)	3 (7)	0 (0)
Other	1 (2.7)	2 (5.6)	1 (2.3)	0 (0)
**Physio intervention**				
Kinisiotape	6 (16.2)	5 (13.9)	7 (16.3)	1 (6.3)
Electrical stimulation	23 (62.2)	22 (61.1)	29 (67.4)	8 (50)
Massage	22 (59.5)	19 (52.8)	27 (62.8)	6 (37.5)
Ultrasound	16 (43.2)	9 (25)	19 (44.2)	2 (12.5)
Ice	19 (51.4)	16 (44.4)	18 (41.9)	5 (31.3)
Kinesiotherapy exercises	17 (45.9)	19 (52.8)	24 (55.8)	2 (12.5)
Other	3 (8.1)	1 (2.8)	5 (11.6)	1 (6.3)
**Injury to the same body location within the preceding year (2021–2022)**				
Yes	16 (39)	13 (31.7)	14 (26.9)	6 (26.1)
No	25 (61)	28 (68.3)	38 (73.1)	17 (73.9)
**Injury to the same body location within the current season (2022–2023) (recurrent injury)**				
Yes	9 (22)	8 (19.5)	11 (21.2)	4 (17.4)
No	32 (78)	33 (80.5)	41 (78.8)	19 (82.6)
**Happened**				
Within 2 months after returning from the index injury	4 (9.8)	7 (17.1)	8 (15.4)	3 (13)
2–12 months after returning from the index injury	4 (9.8)	0 (0)	3 (5.8)	1 (4.3)
>12 months after returning from the index injury	3 (7.3)	1 (2.4)	0 (0)	1 (4.3)
**Additional injury/ies during the 2022–2023 season**				
Yes	12 (29.3)	12 (29.3)	18 (34.6)	7 (30.4)
No	29 (70.7)	29 (70.7)	34 (65.4)	16 (69.6)

DF = defender; FW = forward; MF = midfielder; GK = goalkeeper. * Participants could choose up to two answers.

**Table 4:** Additional Injuries during 2022–23 season.

	DF	FW	MF	GK
	N (%)	N (%)	N (%)	N (%)
**Additional Injury/ies during the 2022–23 season**				
Yes	12 (29.3)	12 (29.3)	18 (34.6)	7 (30.4)
No	29 (70.7)	29 (70.7)	34 (65.4)	16 (69.6)
**Additional injuries occurred**				
During a match	8 (66.7)	5 (41.7)	12 (66.7)	3 (42.9)
During a training session	3 (25)	6 (50)	4 (22.2)	2 (28.6)
Don’t know	1 (8.3)	1 (8.3)	2 (11.1)	2 (28.6)
**Removal from play**				
Immediately	4 (33.3)	6 (50)	9 (50)	1 (14.3)
Later	5 (41.7)	2 (16.7)	4 (22.2)	2 (28.6)
Not at all	3 (25)	4 (33.3)	5 (27.8)	4 (57.1)
**Stop football due to injury (days)**				
0 days	1 (8.3)	2 (16.7)	1 (5.6)	2 (28.6)
1–3 days	2 (16.7)	1 (8.3)	4 (22.2)	1 (14.3)
4–7 days	3 (25)	3 (25)	5 (27.8)	3 (42.9)
8–28 days	6 (50)	3 (25)	6 (33.3)	1 (14.3)
29–90 days	0 (0)	3 (25)	2 (11.1)	0 (0)
**Visit a healthcare professional** **—rehabilitation specialist**				
Yes	9 (75)	10 (83.3)	15 (83.3)	5 (71.4)
No	3 (25)	2 (16.7)	3 (16.7)	2 (28.6)
Kind of healthcare professional *				
Doctor	1 (11.1)	1 (10)	3 (20)	2 (40)
Physiotherapist	2 (22.2)	1 (10)	4 (26.7)	1 (20)
Other	9 (100)	10 (100)	11 (73.3)	4 (80)
Treatment followed *				
Physiotherapy	9 (100)	9 (90)	12 (80)	3 (60)
Rest	6 (66.7)	6 (60)	9 (60)	2 (40)
Medication	5 (55.6)	1 (10)	2 (13.3)	1 (20)
Injections	0 (0)	0 (0)	1 (6.7)	0 (0)
Surgery	0 (0)	0 (0)	1 (6.7)	0 (0)
Other	2 (22.2)	1 (10)	0 (0)	0 (0)
**Physio Intervension**				
Kinesiotape	1 (11.1)	1 (10)	3 (20)	1 (20)
Εlectrical stimulation	8 (88.9)	5 (50)	8 (53.3)	3 (60)
Massage	5 (55.6)	4 (40)	9 (60)	2 (40)
Ultrasound	1 (11.1)	3 (30)	6 (40)	2 (40)
Ice	4 (44.4)	3 (30)	9 (60)	2 (40)
Kinesiotherapy-Exercises	6 (66.7)	5 (50)	7 (46.7)	0 (0)
Other	0 (0)	1 (10)	2 (13.3)	1 (20)

DF = Defenders; FW = Forwards; MF = Midfielders; GK = Goalkeepers. * Participants could choose up to two answers.

**Table 5:** Players’ awareness and application of specific injury prevention exercises across playing positions during the season.

	Knowledge		Use for Injury Prevention			
N = 222	Nordic Hamstring Exercise					
	Yes	No	In-Season	Pre-Season	Both	None
DF	56 (88.9)	7 (11.1)	2 (3.2)	19 (30.2)	31 (49.2)	11 (17.5)
FW	51 (83.6)	10 (16.4)	1 (1.6)	18 (29.5)	26 (42.6)	16 (26.2)
MF	61 (88.4)	8 (11.6)	2 (2.9)	24 (34.8)	29 (42)	14 (20.3)
GK	28 (96.6)	1 (3.4)	2 (6.9)	9 (31)	15 (51.7)	3 (10.3)
	**Reverse Nordic**					
DF	46 (73)	17 (27)	0 (0)	19 (30.2)	21 (33.3)	23 (36.5)
FW	37 (60.7)	24 (39.3)	1 (1.6)	15 (24.6)	20 (32.8)	25 (41)
MF	50 (72.5)	19 (27.5)	2 (2.9)	19 (27.5)	21 (30.4)	27 (39.1)
GK	22 (75.9)	7 (24.1)	1 (3.4)	5 (17.2)	12 (41.4)	11 (37.9)
	**Lunges**					
DF	63 (100)	0 (0)	7 (11.1)	11 (17.5)	44 (69.8)	1 (1.6)
FW	59 (96.7)	2 (3.3)	7 (11.5)	10 (16.4)	39 (63.9)	5 (8.2)
MF	63 (91.3)	6 (8.7)	4 (5.8)	16 (23.2)	45 (65.2)	4 (5.8)
GK	29 (100)	0 (0)	1 (3.4)	6 (20.7)	21 (72.4)	1 (3.4)
	**Side plank exercise**					
DF	60 (95.2)	3 (4.8)	3 (4.8)	13 (20.6)	44 (69.8)	3 (4.8)
FW	57 (93.4)	4 (6.6)	4 (6.6)	16 (26.2)	34 (55.7)	7 (11.5)
MF	67 (97.1)	2 (2.9)	2 (2.9)	13 (18.8)	49 (71)	5 (7.2)
GK	28 (96.6)	1 (3.4)	2 (6.9)	4 (13.8)	22 (75.9)	1 (3.4)
	**Front plank exercise**					
DF	63 (100)	0 (0)	3 (4.8)	14 (22.2)	45 (71.4)	1 (1.6)
FW	59 (96.7)	2 (3.3)	4 (6.6)	15 (24.6)	36 (59)	6 (9.8)
MF	65 (94.2)	4 (5.8)	1 (1.4)	14 (20.3)	50 (72.5)	4 (5.8)
GK	29 (100)	0 (0)	2 (6.9)	4 (13.8)	22 (75.9)	1 (3.4)
	**Copenhagen adductor exercise**					
DF	44 (69.8)	19 (30.2)	2 (3.2)	12 (19)	26 (41.3)	23 (36.5)
FW	52 (85.2)	9 (14.8)	5 (8.2)	18 (29.5)	22 (36.1)	16 (26.2)
MF	52 (75.4)	17 (24.6)	4 (5.8)	14 (20.3)	23 (33.3)	28 (40.6)
GK	27 (93.1)	2 (6.9)	2 (6.9)	6 (20.7)	14 (48.3)	7 (24.1)

DF = defender; FW = forward; MF = midfielder; GK = goalkeeper.

## Data Availability

The data presented in this study are available on request from the corresponding author.

## Supplementary Materials

The following supporting information can be downloaded at: https://www.mdpi.com/article/10.3390/jcm14176320/s1, File S1: Distribution of injuries among amateur football players during the 2022–2023 Season; File S2: Injury types, body locations and mechanisms with corresponding injury rates among amateur football players in Greece; File S3: Injury types, body locations and mechanisms with corresponding injury rates of additional injuries among amateur football players in Greece.

## Abbreviations

The following abbreviations are used in this manuscript:

DF	Defenders
FW	Forwards
MF	Midfielders
GK	Goalkeepers
