# Supplementary material for: Injury Pattern According to Player Position in Male Amateur Football Players in Greece: A Retrospective Study
Source: J Clin Med. 2025 Sep 7;14(17):6320. doi: 10.3390/jcm14176320 (PMC12428947; doi:10.3390/jcm14176320)
Supplement: Supplementary file 1 [file jcm-14-06320-s001.zip › Suppl Material File S1.pdf]

Supplementary Material S1

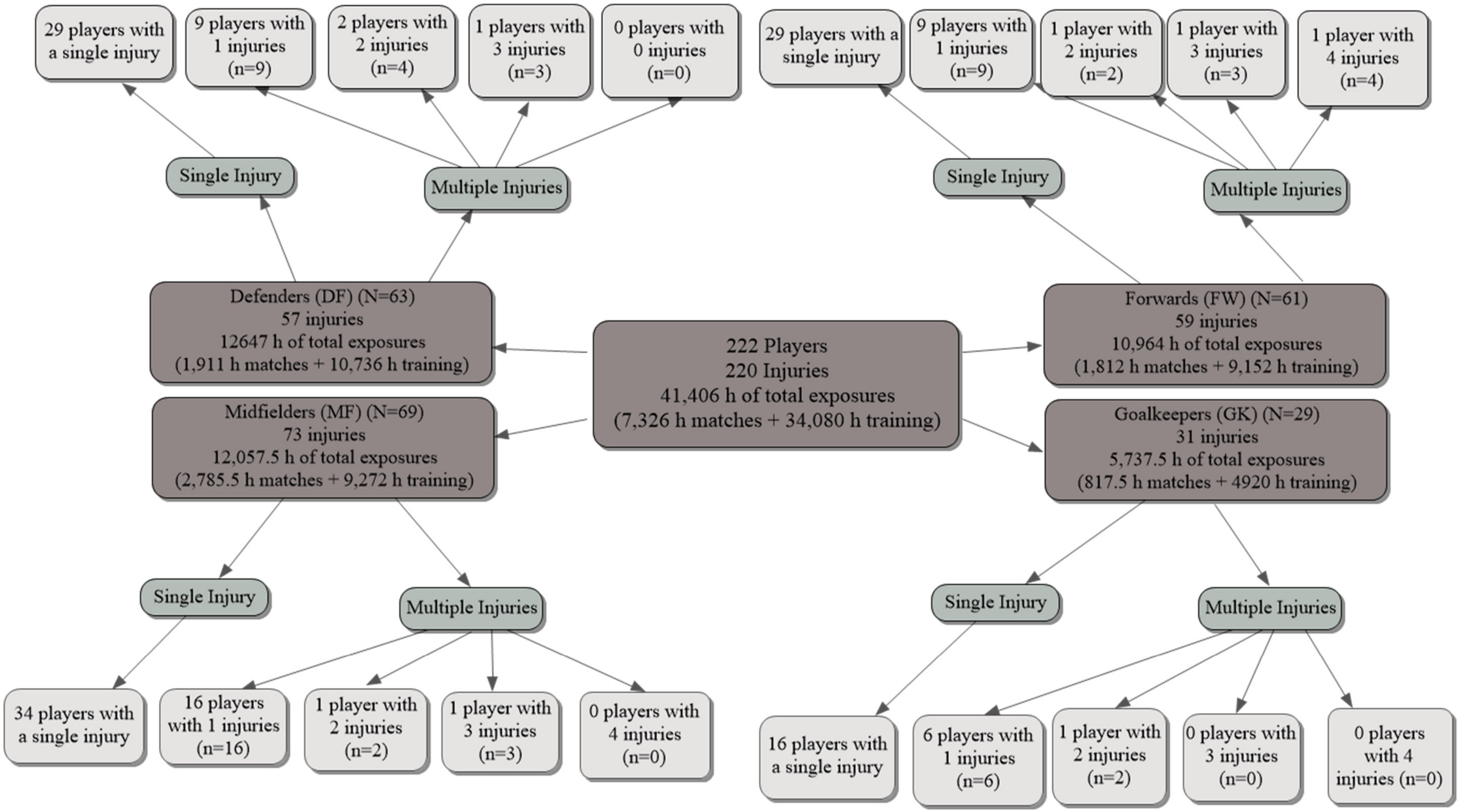

Figure S1 Distribution of injuries among amateur football players during the 2022–23 Season.
