# Supplementary material for: Injury Pattern According to Player Position in Male Amateur Football Players in Greece: A Retrospective Study
Source: J Clin Med. 2025 Sep 7;14(17):6320. doi: 10.3390/jcm14176320 (PMC12428947; doi:10.3390/jcm14176320)
Supplement: Supplementary file 1 [file jcm-14-06320-s001.zip › Suppl Material File S2.pdf]

Supplementary Material S2

Table S1. Injury types, body locations and mechanisms with corresponding injury rates among amateur football players in Greece.

	DF			FW			MF			GK		
	N(%)	IR$	95%CI	N(%)	IR$	95%CI	N(%)	IR$	95%CI	N(%)	IR$	95%CI
**Body location**												
HD	2 (4.9)	0.16	-0.06, 0.38	1 (2.4)	0.09	-0.09, 0.27	0 (0)	0	0, 0	0 (0)	0	0, 0
SH/CV	3 (7.3)	0.24	-0.03, 0.51	0 (0)	0	0, 0	2 (3.8)	0.17	-0.06, 0.4	4 (17.4)	0.7	0.01, 1.38
EL	0 (0)	0	0, 0	0 (0)	0	0, 0	0 (0)	0	0, 0	1 (4.3)	0.17	-0.17, 0.52
WR/FG/TH	1 (2.4)	0.08	-0.08, 0.23	0 (0)	0	0, 0	0 (0)	0	0, 0	2 (8.7)	0.35	-0.13, 0.83
BK	0 (0)	0	0. 0	0 (0)	0	0, 0	1 (1.9)	0.08	-0.08, 0.25	0 (0)	0	0, 0
ABD	1 (2.4)	0.08	-0.08, 0.23	6 (14.6)	0.55	0.11, 0.99	3 (5.8)	0.25	-0.03, 0.53	0 (0)	0	0, 0
LBK	1 (2.4)	0.08	-0.08, 0.23	1 (2.4)	0.09	-0.09, 0.27	1 (1.9)	0.08	-0.08, 0.25	1 (4.3)	0.17	-0.17, 0.52
PL	0 (0)	0	0, 0	0 (0)	0	0, 0)	0 (0)	0	0, 0	0 (0)	0	0, 0
HP/GR	2 (4.9)	0.16	-0.06, 0.38	6 (14.6)	0.55	0.11, 0.99	7 (13.5)	0.58	0.15, 1.01	1 (4.3)	0.17	-0.17, 0.52
THA – QUAD	4 (9.8)	0.32	0.01, 0.63	3 (7.3)	0.27	-0.04, 0.58	4 (7.7)	0.33	0.01, 0.66	0 (0)	0	0, 0
THP – HS	10 (24.4)	0.79	0.3, 1.28	5 (12.2)	0.46	0.06, 0.86	7 (13.5)	0.58	0.15, 1.01	5 (21.7)	0.87	0.11, 1.64
THI – ADD	3 (7.3)	0.24	-0.03, 0.51	7 (17.1)	0.64	0.17, 1.11	9 (17.3)	0.75	0.26, 1.23	4 (17.4)	0.7	0.01, 1.38
KN	4 (9.8)	0.32	0.01, 0.63	5 (12.2)	0.46	0.06, 0.86	12 (23.1)	1	0.43, 1.56	1 (4.3)	0.17	-0.17, 0.52
TBA	0 (0)	0	0, 0)	0 (0)	0	0, 0	0 (0)	0	0, 0	1 (4.3)	0.17	-0.17, 0.52
TBP – SH. AT	2 (4.9)	0.16	-0.06, 0.38	1 (2.4)	0.09	-0.09, 0.27	2 (3.8)	0.17	-0.06, 0.4	0 (0)	0	0, 0
FT/TO	8 (19.5)	0.63	0.19, 1.07	5 (12.2)	0.46	0.06, 0.86	4 (7.7)	0.33	0.01, 0.66	2 (8.7)	0.35	-0.13, 0.83
**Type of injury***												
Sprain	8 (19.5)	0.63	0.19, 1.07	6 (14.6)	0.55	0.11, 0.99	6 (11.5)	0.5	0.1, 0.9	4 (17.4)	0.7	0.01, 1.38
Strain	16 (39)	1.27	0.65, 1.89	20 (48.8)	1.82	1.02, 2.62	24 (46.2)	1.99	1.19, 2.79	7 (30.4)	1.22	0.32, 2.12
Tendinopathy	2 (4.9)	0.16	-0.06, 0.38	1 (2.4)	0.09	-0.09, 0.27	2 (3.8)	0.17	-0.06, 0.4	1 (4.3)	0.17	-0.17, 0.52
Contusion	4 (9.8)	0.32	0.01, 0.63	2 (4.9)	0.18	-0.07, 0.44	2 (3.8)	0.17	-0.06, 0.4	4 (17.4)	0.7	0.01, 1.38
Fracture	1 (2.4)	0.08	-0.08, 0.23	1 (2.4)	0.09	-0.09, 0.27	4 (7.7)	0.33	0.01, 0.66	3 (13)	0.52	-0.07, 1.11
Dislocation	2 (4.9)	0.16	-0.06, 0.38	3 (7.3)	0.27	-0.04, 0.58	2 (3.8)	0.17	-0.06, 0.4	2 (8.7)	0.35	-0.13, 0.83
Other	3 (7.3)	0.24	-0.03, 0.51	1 (2.4)	0.09	-0.09, 0.27	7 (13.5)	0.58	0.15, 1.01	(0)	0	0, 0
Overuse	5 (12.2)	0.4	0.05, 0.74	7 (17.1)	0.64	0.17, 1.11	5 (9.6)	0.41	0.05, 0.78	2 (8.7)	0.35	-0.13, 0.83
**Injury mechanism**												
Tackling	4 (9.8)	0.32	0.01, 0.63	2 (4.9)	0.18	-0.07, 0.44	0 (0)	0	0, 0	0 (0)	0	0, 0
Tackled by opponents	1 (2.4)	0.08	-0.08, 0.23	2 (4.9)	0.18	-0.07, 0.44	2 (3.8)	0.17	-0.06, 0.4	2 (8.7)	0.35	-0.13, 0.83
Running/sprint	14 (34.1)	1.11	0.53, 1.69	17 (41.5)	1.55	0.81, 2.29	15 (28.8)	1.24	0.61, 1.87	3 (13)	0.52	-0.07, 1.11
Falling	0 (0)	0	0, 0	3 (7.3)	0.27	-0.04, 0.58	0 (0)	0	0, 0	5 (21.7)	0.87	0.11, 1.64
Shooting	2 (4.9)	0.16	-0.06, 0.38	4 (9.8)	0.36	0.01, 0.72	2 (3.8)	0.17	-0.06, 0.4	3 (13)	0.52	-0.07, 1.11
Dribbling	0 (0)	0	0, 0	3 (7.3)	0.27	-0.04, 0.58	1 (1.9)	0.08	-0.08, 0.25	0 (0)	0	0, 0
Locking	2 (4.9)	0.16	-0.06, 0.38	1 (2.4)	0.09	-0.09, 0.27	2 (3.8)	0.17	-0.06, 0.4	4 (17.4)	0.7	0.01, 1.38
Jumping	2 (4.9)	0.16	-0.06, 0.38	2 (4.9)	0.18	-0.07, 0.44	3 (5.8)	0.25	-0.03, 0.53	1 (4.3)	0.17	-0.17, 0.52
Landing	1 (2.4)	0.08	-0.08, 0.23	4 (9.8)	0.36	0.01, 0.72	1 (1.9)	0.08	-0.08, 0.25	2 (8.7)	0.35	-0.13, 0.83
Heading	1 (2.4)	0.08	-0.08, 0.23	1 (2.4)	0.09	-0.09, 0.27	0 (0)	0	0, 0	0 (0)	0	0, 0
Turning/rotation	2 (4.9)	0.16	-0.06, 0.38	2 (4.9)	0.18	-0.07, 0.44	5 (9.6)	0.41	0.05, 0.78	0 (0)	0	0, 0
Collision	3 (7.3)	0.24	-0.03, 0.51	4 (9.8)	0.36	0.01, 0.72	6 (11.5)	0.5	0.1, 0.9	4 (17.4)	0.7	0.01, 1.38
Overuse	6 (14.6)	0.47	0.09, 0.85	8 (19.5)	0.73	0.22, 1.24	5 (9.6)	0.41	0.05, 0.78	1 (4.3)	0.17	-0.17, 0.52
Unknown mechanism	3 (7.3)	0.24	-0.03, 0.51	6 (14.6)	0.55	0.11, 0.99	6 (11.5)	0.5	0.1, 0.9	1 (4.3)	0.17	-0.17, 0.52
Other	5 (12.2)	0.4	0.05, 0.74	2 (4.9)	0.18	-0.07, 0.44	8 (15.4)	0.66	0.2, 1.12	2 (8.7)	0.35	-0.13, 0.83

DF = Defenders; FW = Forwards; MF = Midfielders; GK = Goalkeepers; HD = Head; SH/CV = Shoulder / Clavicle; EL = Elbow; WR/FG/TH = Wrist / Finger(s) / Thumb; BK = Back; ABD = Abdomen; LBK = Lower back; PL = Pelvis; HP/GR = Hip/groin; THA – QUAD = Thigh (Anterior) – Quadriceps; THP – HS = Thigh (Posterior) – Hamstrings; THI – ADD = Thigh (Inner) – Adductors; KN = Knee; TBA = Tibia (Anterior); TBP – SH. AT = Tibia (Posterior) – Shank. Achilles tendon; FT/TO = Foot/Toe; IR=Injury Rate
* Participants could chose type up to two answers. $number of injuries per 1,000 hours of total exposure.
