# Supplementary material for: Injury Pattern According to Player Position in Male Amateur Football Players in Greece: A Retrospective Study
Source: J Clin Med. 2025 Sep 7;14(17):6320. doi: 10.3390/jcm14176320 (PMC12428947; doi:10.3390/jcm14176320)
Supplement: Supplementary file 1 [file jcm-14-06320-s001.zip › Suppl Material File S3.pdf]

# Supplementary Material S3

Table S2. Injury types, body locations and mechanisms with corresponding injury rates of additional injuries among amateur football players in Greece.

| | DF | | | FW | | | MF | | | GK | | |
|---|---|---|---|---|---|---|---|---|---|---|---|---|
| | N(%) | IR[$] | 95%CI | N(%) | IR[$] | 95%CI | N(%) | IR[$] | 95%CI | N(%) | IR[$] | 95%CI |
| **Body location** | | | | | | | | | | | | |
| HD | 2 (16.7) | 0.16 | -0.06, 0.38 | 0 (0) | 0 | 0, 0 | 0 (0) | 0 | 0, 0 | 1 (14.3) | 0.17 | -0.17, 0.52 |
| FC | 0 (0) | 0 | 0, 0 | 0 (0) | 0 | 0, 0 | 1 (5.6) | 0.08 | -0.08. 0.25 | 0 (0) | 0 | 0, 0 |
| NK | 0 (0) | 0 | 0, 0 | 0 (0) | 0 | 0, 0 | 0 (0) | 0 | 0, 0 | 0 (0) | 0 | 0, 0 |
| SH/CV | 0 (0) | 0 | 0, 0 | 0 (0) | 0 | 0, 0 | 0 (0) | 0 | 0, 0 | 1 (14.3) | 0.17 | -0.17, 0.52 |
| EL | 0 (0) | 0 | 0, 0 | 0 (0) | 0 | 0, 0 | 0 (0) | 0 | 0, 0 | 0 (0) | 0 | 0, 0 |
| WR/FG/TH | 1 (8.3) | 0.08 | -0.08, 0.23 | 1 (8.3) | 0.09 | -0.09, 0.27 | 0 (0) | 0 | 0, 0 | 0 (0) | 0 | 0, 0 |
| BK | 0 (0) | 0 | 0, 0 | 0 (0) | 0 | 0, 0 | 0 (0) | 0 | 0, 0 | 0 (0) | 0 | 0, 0 |
| ABD | 0 (0) | 0 | 0, 0 | 1 (8.3) | 0.09 | -0.09, 0.27 | 0 (0) | 0 | 0, 0 | 1 (14.3) | 0.17 | -0.17, 0.52 |
| LBK | 0 (0) | 0 | 0, 0 | 1 (8.3) | 0.09 | -0.09, 0.27 | 2 (11.1) | 0.17 | -0.06, 0.4 | 0 (0) | 0 | 0, 0 |
| PL | 0 (0) | 0 | 0, 0 | 1 (8.3) | 0.09 | -0.09, 0.27 | 1 (5.6) | 0.08 | -0.08, 0.25 | 0 (0) | 0 | 0, 0 |
| HP/GR | 2 (16.7) | 0.16 | -0.06, 0.38 | 1 (8.3) | 0.09 | -0.09, 0.27 | 1 (5.6) | 0.08 | -0.08, 0.25 | 0 (0) | 0 | 0, 0 |
| THA – QUAD | 1 (8.3) | 0.08 | -0.08, 0.23 | 2 (16.7) | 0.18 | -0.07, 0.44 | 3 (16.7) | 0.25 | -0.03, 0.53 | 1 (14.3) | 0.17 | -0.17, 0.52 |
| THP – HS | 5 (41.7) | 0.4 | 0.05, 0.74 | 1 (8.3) | 0.09 | -0.09, 0.27 | 3 (16.7) | 0.25 | -0.03, 0.53 | 2 (28.6) | 0.35 | -0.13, 0.83 |
| THI – ADD | 0 (0) | 0 | 0, 0 | 3 (25) | 0.27 | -0.04, 0.58 | 4 (22.2) | 0.33 | 0.01, 0.66 | 2 (28.6) | 0.35 | -0.13, 0.83 |
| KN | 1 (8.3) | 0.08 | -0.08, 0.23 | 2 (16.7) | 0.18 | -0.07, 0.44 | 3 (16.7) | 0.25 | -0.03, 0.53 | 0 (0) | 0 | 0, 0 |
| TBA | 1 (8.3) | 0.08 | -0.08, 0.23 | 1 (8.3) | 0.09 | -0.09, 0.27 | 0 (0) | 0 | 0, 0 | 0 (0) | 0 | 0, 0 |
| TBP – SH, AT | 1 (8.3) | 0.08 | -0.08, 0.23 | 0 (0) | 0 | 0, 0 | 1 (5.6) | 0.08 | -0.08, 0.25 | 0 (0) | 0 | 0, 0 |
| FT/TO | 2 (16.7) | 0.16 | -0.06, 0.38 | 4 (33.3) | 0.36 | 0.01, 0.72 | 2 (11.1) | 0.17 | -0.06, 0.4 | 0 (0) | 0 | 0, 0 |
| **Type of injury*** | | | | | | | | | | | | |
| Sprain | 2 (16.7) | 0.16 | -0.06, 0.38 | 4 (33.3) | 0.36 | 0.01, 0.72 | 3 (16.7) | 0.25 | -0.03, 0.53 | 0 (0) | 0 | 0, 0 |
| Strain | 7 (58.3) | 0.55 | 0.14, 0.96 | 5 (41.7) | 0.46 | 0.06, 0.86 | 9 (50) | 0.75 | 0.26, 1.23 | 4 (57.1) | 0.7 | 0.01, 1.38 |
| Tendinopathy | 0 (0) | 0 | 0, 0 | 2 (16.7) | 0.18 | -0.07, 0.44 | 0 (0) | 0 | 0, 0 | 1 (14.3) | 0.17 | -0.17, 0.52 |
| Contusion | 1 (8.3) | 0.08 | -0.08, 0.23 | 0 (0) | 0 | 0, 0 | 0 (0) | 0 | 0, 0 | 0 (0) | 0 | 0, 0 |
| Fracture | 1 (8.3) | 0.08 | -0.08, 0.23 | 1 (8.3) | 0.09 | -0.09, 0.27 | 1 (5.6) | 0.08 | -0.08, 0.25 | 0 (0) | 0 | 0, 0 |
| Dislocation | 0 (0) | 0 | 0, 0 | 0 (0) | 0 | 0, 0 | 0 (0) | 0 | 0, 0 | 0 (0) | 0 | 0, 0 |
| Other | 3 (25) | 0.24 | -0.03, 0.51 | 1 (8.3) | 0.09 | -0.09, 0.27 | 2 (11.1) | 0.17 | -0.06, 0.4 | 2 (28.6) | 0.35 | -0.13, 0.83 |
| Overuse | 1 (8.3) | 0.08 | -0.08, 0.23 | 1 (8.3) | 0.09 | -0.09, 0.27 | 4 (22.2) | 0.33 | 0.01, 0.66 | 0 (0) | 0 | 0, 0 |
| **Injury mechanism** | | | | | | | | | | | | |
| Tackling | 2 (16.7) | 0.16 | -0.06, 0.38 | 0 (0) | 0 | 0, 0 | 1 (5.6) | 0.08 | -0.08. 0.25 | 0 (0) | 0 | 0. 0 |
| Tackled by opponents | 0 (0) | 0 | 0, 0 | 2 (16.7) | 0.18 | -0.07, 0.44 | 1 (5.6) | 0.08 | -0.08. 0.25 | 0 (0) | 0 | 0. 0 |
| Running/sprint | 6 (50) | 0.47 | 0.09, 0.85 | 2 (16.7) | 0.18 | -0.07, 0.44 | 5 (27.8) | 0.41 | 0.05. 0.78 | 1 (14.3) | 0.17 | -0.17. 0.52 |
| Falling | 1 (8.3) | 0.08 | -0.08, 0.23 | 0 (0) | 0 | 0, 0 | 1 (5.6) | 0.08 | -0.08. 0.25 | 0 (0) | 0 | 0. 0 |
| Shooting | 1 (8.3) | 0.08 | -0.08, 0.23 | 2 (16.7) | 0.18 | -0.07, 0.44 | 2 (11.1) | 0.17 | -0.06. 0.4 | 2 (28.6) | 0.35 | -0.13. 0.83 |
| Dribbling | 0 (0) | 0 | 0, 0 | 1 (8.3) | 0.09 | -0.09, 0.27 | 0 (0) | 0 | 0. 0 | 0 (0) | 0 | 0. 0 |
| Locking | 2 (16.7) | 0.16 | -0.06, 0.38 | 0 (0) | 0 | 0, 0 | 0 (0) | 0 | 0. 0 | 0 (0) | 0 | 0. 0 |
| Jumping | 0 (0) | 0 | 0, 0 | 0 (0) | 0 | 0, 0 | 1 (5.6) | 0.08 | -0.08. 0.25 | 0 (0) | 0 | 0. 0 |
| Landing | 0 (0) | 0 | 0, 0 | 0 (0) | 0 | 0, 0 | 0 (0) | 0 | 0. 0 | 1 (14.3) | 0.17 | -0.17. 0.52 |
| Heading | 2 (16.7) | 0.16 | -0.06, 0.38 | 0 (0) | 0 | 0, 0 | 0 (0) | 0 | 0. 0 | 0 (0) | 0 | 0. 0 |
| Turning/rotation | 0 (0) | 0 | 0, 0 | 1 (8.3) | 0.09 | -0.09, 0.27 | 2 (11.1) | 0.17 | -0.06. 0.4 | 0 (0) | 0 | 0. 0 |
| Collision | 1 (8.3) | 0.08 | -0.08, 0.23 | 2 (16.7) | 0.18 | -0.07, 0.44 | 1 (5.6) | 0.08 | -0.08. 0.25 | 1 (14.3) | 0.17 | -0.17. 0.52 |
| Overuse | 1 (8.3) | 0.08 | -0.08, 0.23 | 1 (8.3) | 0.09 | -0.09, 0.27 | 2 (11.1) | 0.17 | -0.06. 0.4 | 0 (0) | 0 | 0. 0 |
| Unknown mechanism | 1 (8.3) | 0.08 | -0.08, 0.23 | 2 (16.7) | 0.18 | -0.07, 0.44 | 3 (16.7) | 0.25 | -0.03. 0.53 | 2 (28.6) | 0.35 | -0.13. 0.83 |
| Other | 0 (0) | 0 | 0, 0 | 0 (0) | 0 | 0, 0 | 0 (0) | 0 | 0. 0 | 0 (0) | 0 | 0. 0 |

DF = Defenders; FW = Forwards; MF = Midfielders;GK = Goalkeepers; HD = Head; FC = Face; NK = Neck; SH/CV = Shoulder / Clavicle; EL = Elbow; WR/FG/TH = Wrist / Finger(s) / Thumb; BK = Back; ABD = Abdomen; LBK = Lower back; PL = Pelvis; HP/GR = Hip/groin; THA – QUAD = Thigh (Anterior) – Quadriceps; THP – HS = Thigh (Posterior) – Hamstrings; THI – ADD = Thigh (Inner) – Adductors; KN = Knee; TBA = Tibia (Anterior); TBP – SH, AT = Tibia (Posterior) – Shank, Achilles tendon; FT/TO = Foot/Toe; IR=Injury Rate * Participants could chose type up to two answers. [$]number of injuries per 1,000 hours of total exposure.
